# Highlights of Biosafety and Biosecurity Month (BBM)at the AIMST University and Perspectives on Biorisk Management

**DOI:** 10.6026/97320630015568

**Published:** 2019-09-07

**Authors:** Subhash Janardhan Bhore

**Affiliations:** 1 Chairman, AIMST University Institutional Biosafety Committee; 2 2Department of Biotechnology, Faculty of Applied Sciences, AIMST University, Bedong-Semeling Road, 08100 Bedong, Semeling, Kedah Darul Aman, Malaysia

**Keywords:** biorisk, biosafety, biosecurity, biotechnology, microbiology, sustainability

## Abstract

The innovations and developments in microbiology, biomedical sciences, and biotechnology come along with the challenges of biological risk (biorisk). Biorisk is defined as the "combination of the probability of occurrence of harm and the severity of that harm where the source of harm is a biological agent or toxin." Biorisk is a borderless challenge to the global community. Hence, all universities, colleges, centers of bio-excellence, and institutions of higher learning can and should do their bit to educate technical members, academicians, students and stakeholders (LASS) for the efficient and comprehensive biorisk management (BRM) for our and future generations safety and sustainability.

## Abbreviations:

BBM, Biosafety and biosecurity month; BRM, Biorisk management; CD-ERT, Civil defence emergency response team; DBM, Department of Biosafety, Malaysia; IBC, Institutional biosafety committee; IHL, Institutions of higher learning; IKM, Institut Kimia Malaysia; JPAM, Department of civil defence Malaysia; KIMIA, Department of chemistry Malaysia; LASS, Laboratorians, academicians, students and stakeholders; LMOs, Living modified organisms; OSHEC, Occupational safety, health, and environment committee; PASS, pull, aim, squeeze, and sweep.

## Comments and Views:

Innovations and breakthroughs in various domains of microbiology, biomedical sciences, and biotechnology are significantly boosting practices in agriculture, health care, and the pharmaceutical industry [1]. Advancements in biotechnology and research on biological agents and their derivatives necessitate the effective implementation of biorisk management (BRM) at all levels, bearing in mind the potential of dual biological use and ability of microorganisms to move rapidly around the world in a very short time [2]. Hence, for the efficient and comprehensive BRM, biosafety and biosecurity awareness among laboratorians, academicians, students, and stakeholders (LASS) is essential [3, 4].

The institutional biosafety committee (IBC) of the AIMST University [5] and the occupational safety, health, and environment committee (OSHEC) [6] had observed April-2019 as the 'Biosafety and Biosecurity Month (BBM)' aimed to promote the awareness about the importance of biosafety and biosecurity among its LASS [7]. The theme of BBM was "beyond the laboratories: increasing the visibility of biosafety and biosecurity". It was designed as per the BBM theme of ABSA International, the American Biological Safety Association [7]. Events such as (1) launching of institutional biosafety committee (IBC) website for university, (2) biosafety training workshop, (3) a 'symposium on chemical, biological and health safety', (4) briefing and a mock fire drill, and (5) 'poster presentation competition' were organized to promote awareness among university's LASS.

IBC website was launched on the 5th April 2019 by Emeritus Professor Dr. Harcharan Singh (Vice-Chancellor and Chief Executive of AIMST University) [5]. While launching the IBC website, he emphasized the importance of our commitment for ensuring biosafety and biosecurity in compliance to the Biosafety Act-2007 of Malaysia [8] and international standards and or guidelines [9]. We further conducted the Biosafety training workshop on the 9th April 2019 in collaboration with Department of Biosafety, Malaysia (DBM) [10].

Dr Anita Anthonysamy from DBM highlighted that the Biosafety Act-2007 in Malaysia provides checks and balances for products of modern biotechnology, such as living modified organisms (LMOs) such that they do not pose unacceptable risks (to human, animals and the environment), for reaping the real benefits of modern biotechnology towards national growth and development.

A 'Symposium on Chemical, Biological and Health Safety' held in collaboration with the Department of Chemistry Malaysia (KIMIA) [https://www.kimia.gov.my/v3/en/] and Institut Kimia Malaysia (IKM) [https://www.ikm.org.my/] on the 16th April 2019 at AIMST University [http://www.aimst.edu.my/] helped participants in understanding the elements of chemical safety, and the need of implementing biorisk management system for laboratory safety. A mock 'fire drill and briefing on fire safety' [11] was arranged on the 24th April 2019 at the AIMST University in collaboration with the Department of Civil Defence Malaysia (JPAM) [http://www.civildefence.gov.my/] and Civil Defence Emergency Response Team (CD-ERT). This programme helped hundreds of students and staff members in understanding the four steps ('pull, aim, squeeze, and sweep' (PASS) concept) involved in using a fire extinguisher [12] while combating the fire outbreak with practical drill sessions. A poster presentation competition on the 30th April 2019, helped a few dozen students to promote the importance of biosafety and biosecurity at the AIMST University. Hundreds of biotechnology and biomedical students benefited from the posters' session on biosafety, biosecurity, and BRM.

**Figure 1:**
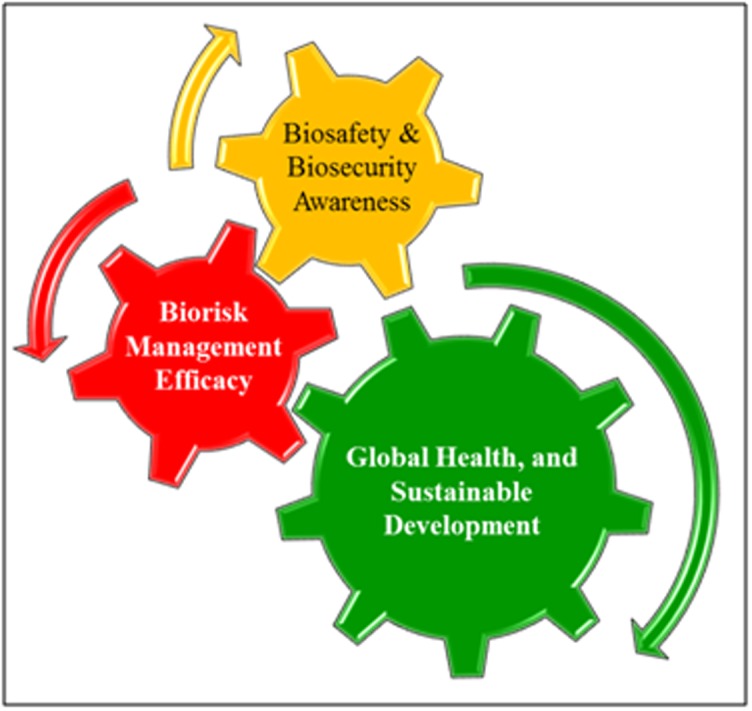
Biosafety and biosecurity awareness will enhance the comprehensive biorisk management, and as a result, it will help in the safeguarding of human, animal and environmental health (global health) issues for sustainable development.

**Figure 2:**
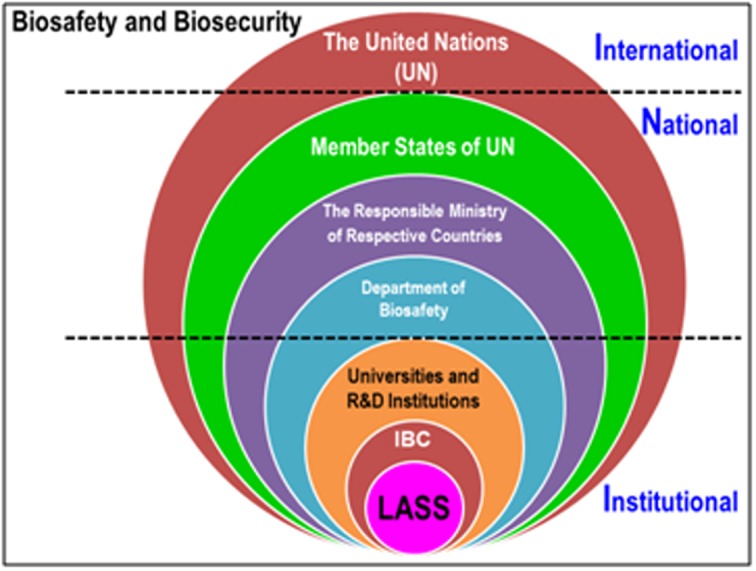
The schematic diagram showing the importance of LASS (laboratorians, academicians, students, and stakeholders) in practicing biosafety and biosecurity (Biorisk Management) for the institutional (local), national, and international (global) safety and security. R and D, research and development; IBC, institutional biosafety committee [6].

Biosafety and biosecurity education through various practical activities helps to make BRM more effective in protecting humans, plants, animals, and the environment (Figure 1). The promotion of awareness about biosafety and biosecurity among LASS and compliance to local and national regulations is essential for the safety and security at the local and global level (Figure 2). However, the onus is on the regulatory agencies, stakeholders, and biorisk management (biosafety and biosecurity) professionals that work in the areas of public health, hospitals, veterinary and animal health, environmental health, related government departments, and academia.

## Conclusion

In brief, the implementation of efficient and comprehensive BRM is essential to protect LASS, community, and the surrounding environment. Biorisk has increased significantly with the advancements in microbiology, biomedical sciences, and biotechnology. For effective BRM, the good practices of biosafety and biosecurity should be promoted beyond laboratories. Observing BBM is a practical approach to promote awareness about the importance of biosafety and biosecurity among LASS. AIMST University was the first to observe BBM. However, all universities, colleges, centers of bio-excellence, and (or) institutions of higher learning (IHL) should promote awareness about the importance of biosafety and biosecurity for effective biorisk management, secure future, and sustainability.
